# First Whole-Genome Sequences of Two Multidrug-Resistant *Acinetobacter baumannii* Strains Isolated from a Moroccan Hospital Floor

**DOI:** 10.1128/genomeA.00298-17

**Published:** 2017-05-04

**Authors:** Tarek Alouane, Jean Uwingabiye, Abdelhay Lemnouer, Lamiaa Lahlou, Meriem Laamarti, Souad Kartti, Oussama Benhrif, Hamza El Misbahi, Mohammed Frikh, Yassine Benlahlou, Fatna Bssaibis, Soukaina El Abbassi, Samia Kabbage, Adil Maleb, Mostafa Elouennass, Azeddine Ibrahimi

**Affiliations:** a Biotechnology Lab (MedBiotech), Rabat Medical and Pharmacy School, Mohammed V University, Rabat, Morocco; b Department of Bacteriology, Mohammed V Military Teaching Hospital, Research Unit, Epidemiology and Bacterial Resistance, Rabat Medical and Pharmacy School, Mohammed V University, Rabat, Morocco

## Abstract

This report describes the whole-genome shotgun sequences of two multidrug-resistant *Acinetobacter baumannii* strains, ABE8_07 and ABE12_M, isolated from a Moroccan hospital floor. These two genome sequences will initiate the study and characterization of the *Acinetobacter baumannii* genome in Morocco.

## GENOME ANNOUNCEMENT

*Acinetobacter baumannii* is a Gram-negative coccobacillus known for its remarkable persistence in hospital environments worldwide; it causes nosocomial infection, and reports have shown that it is potentially resistant to several antibiotics, thus limiting current therapeutic options (1, 2). Previous Moroccan studies have shown that, during the period of 2003 to 2016, this microorganism represented 6.94% of all bacterial infections and 9.6% of all Gram-negative bacilli (3) and that its antibiotic resistance rates increased from 23 to 76% for imipenem, 63 to 86% for ceftazidime, 41 to 52% for amikacin, and 68 to 87% for ciprofloxacin (3, 4).

In this study, we present the draft genome sequences of two multidrug-resistant *A. baumannii* strains, isolated from a Moroccan hospital floor, that demonstrate resistance to various antibiotics, including ticarcillin, piperacillin, ticarcillin-clavulanic acid, piperacillin-tazobactam, ceftazidime, cefepime, imipenem, gentamicin, tobramycin, amikacin, netilmicin, ciprofloxacin, tetracycline, and cotrimoxazole (but not colistin).

Genomic DNA was extracted using a DNA IQ system kit (Promega) and quantified using a NanoVue Plus spectrophotometer (Biochrom). The library of genomic DNA was prepared using a Nextera XT DNA library preparation kit (Illumina), with dual indexing adapters, and sequenced using an Illumina MiSeq sequencer with a 2 × 251-bp paired-end configuration. The sequencing generated 2,843,500 reads for ABE8_07 and 601,494 reads for ABE12_M. The sequences were trimmed and *de novo* assembled using CLC Genomics Workbench version 9.5 (Qiagen). Strain ABE8_07 comprised 187 contigs with a total length of 3,898,083 bp, an *N*_50_ of 57,519 bp, and an average contig length of 20,845 bp, while strain ABE12_M comprised 196 contigs with a total size of 3,902,530 bp, an *N*_50_ of 54,846 bp, and an average contig length of 19,911 bp. The G+C contents for ABE8_07 and ABE12_M were 38.97% and 38.95%, respectively.

The genomes were annotated using Prokka version 1.7 (5) and the Rapid Annotations using Subsystems Technology (RAST) server (6). The annotation of strain ABE8_ 07 detected 3,657 protein-coding genes, 63 tRNA genes, and 6 rRNAs, representing 456 subsystems. The annotation of strain ABE12_M detected 3,669 protein-coding genes, 61 tRNA genes, and 3 rRNA genes, representing 453 subsystems.

Analysis of antimicrobial resistance genes by ResFinder version 2.1 (7) indicated the presence of multiple genes encoding for resistance to β-lactams (*bla*_ADC-25_, *bla*_OXA-23_, *bla*_OXA-66_, and *bla*_TEM-1D_), aminoglycosides [*aph(3′)-VIa*, *aph [3′]-Ic*, *aadA1*, *aacA4*, *strA*, *strB*, and *armA*], fluoroquinolone-aminoglycosides [*aac(6′)Ib-cr*], macrolide-lincosamide-streptogramin [*msr*(E) and *mph*(E)], sulfonamide (*sul1*), phenicol (*catB8*), and tetracycline [*tet*(B)] in both the genomes, except for *aadB*, which was present in ABE12_M only.

### Accession number(s).

This whole-genome shotgun project has been deposited at DDBJ/EMBL/GenBank under the accession numbers FPKN00000000 and FPEF00000000 for strains ABE8_07 and ABE12_M, respectively. The versions described in this article are the first versions, FPKN01000000 and FPEF01000000.
